# Quantifying social contact patterns in Minnesota during stay-at-home social distancing order

**DOI:** 10.1186/s12879-022-07968-1

**Published:** 2023-05-15

**Authors:** Audrey M. Dorélien, Narmada Venkateswaran, Jiuchen Deng, Kelly Searle, Eva Enns, Giovann Alarcon Espinoza, Shalini Kulasingam

**Affiliations:** University of Minnesota, Minneapolis, MN 55455 USA

**Keywords:** Social contacts, Age-structured contact matrices, Stay-at-home orders, SARS-CoV-2

## Abstract

SARS-CoV-2 is primarily transmitted through person-to-person contacts. It is important to collect information on age-specific contact patterns because SARS-CoV-2 susceptibility, transmission, and morbidity vary by age. To reduce the risk of infection, social distancing measures have been implemented. Social contact data, which identify who has contact with whom especially by age and place are needed to identify high-risk groups and serve to inform the design of non-pharmaceutical interventions. We estimated and used negative binomial regression to compare the number of daily contacts during the first round (April–May 2020) of the Minnesota Social Contact Study, based on respondent’s age, gender, race/ethnicity, region, and other demographic characteristics. We used information on the age and location of contacts to generate age-structured contact matrices. Finally, we compared the age-structured contact matrices during the stay-at-home order to pre-pandemic matrices. During the state-wide stay-home order, the mean daily number of contacts was 5.7. We found significant variation in contacts by age, gender, race, and region. Adults between 40 and 50 years had the highest number of contacts. The way race/ethnicity was coded influenced patterns between groups. Respondents living in Black households (which includes many White respondents living in inter-racial households with black family members) had 2.7 more contacts than respondents in White households; we did not find this same pattern when we focused on individual’s reported race/ethnicity. Asian or Pacific Islander respondents or in API households had approximately the same number of contacts as respondents in White households. Respondents in Hispanic households had approximately two fewer contacts compared to White households, likewise Hispanic respondents had three fewer contacts than White respondents. Most contacts were with other individuals in the same age group. Compared to the pre-pandemic period, the biggest declines occurred in contacts between children, and contacts between those over 60 with those below 60.

**Supplementary Information:**

The online version contains supplementary material available at 10.1186/s12879-022-07968-1.

## Introduction

Prior to the availability of COVID-19 vaccines, public health officials and governments were reliant on non-pharmaceutical interventions (NPIs) to control and mitigate the spread of novel infections like SARS-CoV-2. School closures, physical distancing measures, and stay-at-home orders are all examples of NPIs that have been used to reduce the risk of transmission by limiting interpersonal contact. Many infectious disease transmission models that seek to predict the disease trajectory, test the impact of various NPI control measures, and determine optimal vaccination strategies, include age-structured contact patterns as a key model parameter [1–4]. This is because the force of infection is affected by heterogeneity in mixing patterns related to mixing within and between different age groups [5]. It is also important to present age-structured patterns because COVID-19 morbidity and mortality patterns vary by age [6], and this allows us to identify age groups that are at high risk for contracting and transmitting COVID-19 based on reported levels of contact. Very little data was collected prior to the onset of the current COVID-19 pandemic; the existing baseline data are either dated and focused on measuring the mean duration of contacts, focused on small populations or do not provide detailed information for non-household settings [7–9]. The first nationally representative social contact survey of adult respondents took place between August-December 2020 and March–April 2021 [10]. Nevertheless, until recently, researchers modeling SARS-CoV-2 transmission in the U.S. have relied on data from European countries or extrapolated estimates of what U.S. contact patterns may look like [11, 12].

Our study makes many important contributions to the scientific literature on social contact patterns. We analyze data from the Minnesota Social Contact Study (MN SCS), which collected information on all age groups (children and adults) during the pandemic using a representative sample of the Minnesota non-institutional population. Other recent studies conducted in the U.S. focused only on adults (18 years and older) and or did not use a population-representative sample of the US or any state’s population [2, 10]. The intensity and frequency of pandemics are increasing [13]; therefore, it is important to understand the effect of NPIs such as stay-at-home orders on contact and mixing patterns. Consequently, we identify structural (e.g., weekday versus weekend) and socio-demographic factors (e.g., race, region, etc.) that are associated with an elevated number of contacts during the stay-at-home (SAH) order in Minnesota. We quantify the extent to which the SAH order altered the number and pattern of contacts by comparing the MN Round 1 (SAH) matrix with pre-pandemic contact matrices. Specifically, we compare our age-structured mixing patterns with mixing patterns from the United Kingdom (UK POLYMOD) in 2006 [14]. The POLYMOD (Improving Public Health Policy in Europe through Modelling and Economic Evaluation of Interventions for the Control of Infectious Diseases) surveys are the most widely cited social contact surveys; they were conducted in 2006 in eight different European countries. We also compare our findings with a US synthetic contact matrix, based on data from POLYMOD surveys and data on US household composition, labor force participation, school enrollment rates, and population age structure [11]. Finally, we compare the contacts occurring in the home with a matrix generated from American Time Use Survey data [9]. We highlight which age groups had the greatest changes in behavior during the SAH order. Finally, we compare the MN SAH contact patterns and age-structured contact matrix with contact patterns from other movement control orders in other geographic settings.

## Materials and methods

### Ethics statement

Participation in the MN SCS survey was voluntary, informed consent was obtained from all subjects, and from for subject under age 18 consent was obtained from parent /or legal guardian. All analysis was carried out on anonymized data. The study was approved by the University of Minnesota Institutional Review Board PRF (0706S10181). All methods were carried out in accordance with relevant guidelines and regulations.

### Survey population

Round 1 of the Minnesota Social Contact Study (MN SCS) was collected between April 17th and May 17th, 2020. During this period the Emergency Executive Order 20–20, which directed Minnesotans to stay at home, was extended and the stay-at-home order was still in effect [15, 16]. This order mandated that Minnesotans, with the exceptions of essential workers, stay at home and practice physical distancing when in public. Also, any worker who can work from home, including essential workers in the critical sectors, was required to do so. Essential workers were classified according to the Department of Health as those employed in childcare, social work, or administration related to these positions; critical infrastructure; farming; food production, retail, or essential retail; and health care, elder-care, and individual or family care [15]. The definition of an essential worker was broad and encompassed more than 75% of the workforce [17]. The data collection period took place during the start of the first wave of infections in Minnesota. During the data collection period, the test positivity rate for SARS-CoV-2 reached a maximum of 15%, COVID-19 hospitalizations reached 9.8 weekly admissions per 100,000 residents, and there were 396 deaths [18].

### MN SCS weighting

The MN SCS drew its sample from past participants in the 2019 Minnesota Health Access Survey (MNHA) who indicated their willingness to participate in a follow-up survey. Half of this population was randomly selected for Round 1 of the MN SCS. Because the MN SCS draws its sample from the MNHA and can be thought of as a longitudinal sub-sample, we use the MNHA base weights to model the MN SCS base weights. We applied response propensity weighting to adjust for survey nonresponse. Most notably, the Survey of Income and Program Participation and the Medical Expenditure Panel Survey use this method to adjust their weights for attrition between rounds. We used logistical regressions of respondents’ decisions to continue in the sample and a set of demographic variables from the MNHA as covariates to model for respondents’ propensity of attrition [19, 20].These covariates are the age of the MNHA target and respondent, sex of the MNHA target and respondent, the relationship between the MNHA target and the respondent, race and ethnicity, educational level, marital status, homeownership, country of origin, employment status, income, household size, area of residence (i.e., Census’ Public Use Microdata Area), and internet access.

Using these adjusted base weights, we post-stratify the base weights to the estimated population counts, mainly obtained from the 2018 American Community Survey restricted for Minnesota residents only. As in the MNHA, we follow a first post-stratification step for prepaid cell phones (they were oversampled to be more representative because overtime the percentage of responses from cellphones had been increasing) before appending the Landline and Cell frames [21]. Then, we post-stratify the random digit dial (RDD) and address-based (ABS) samples independently using a set of demographic variables. These variables are age, sex, household size, home ownership, race/ethnicity at the household level (e.g., indicating the presence of racial and ethnic groups in the household), children’s presence in the household, highest educational level in the household, household size, internet access, and area of residence (i.e., Census’ Public Use Microdata Area). Finally, we use the effective sample size composite to append both frames in the MN SCS and obtain the final MN SCS weights. In Table 1, we present the unweighted and weighted distribution of the sample based on certain characteristics. The MN SCS Round 1 over sampled non-metro regions. White and Native American households were overrepresented, while Black, Asian, and Hispanic households were under-represented.

**Table 1:** Descriptive statistics showing the distribution of participants and daily contacts by demographic characteristics (N = 2083)

		Weighted	Number of contacts					Unweighted	Number of contacts	
	N	%	Mean	SD	SE	IRR	95% CI	%	Mean	SE
Respondent (child or adult giving contact information)	2083		5.68		0.23	–	–		5.39	0.22
Respondent's five-year age group										
0-4	108	5.79	4.34	3.98	0.38	1.13	[0.73,1.74]	5.18	4.31	0.40
5-9	107	5.68	4.50	3.26	0.32	1.13	[0.74,1.72]	5.14	4.44	0.30
10-14	144	6.22	4.71	2.46	0.21	1.19	[0.81,1.76]	6.91	4.31	0.25
15-19	138	6.72	7.17	13.08	1.11	1.82	[1.00,3.30]	6.63	6.28	1.03
20-24	31	3.50	4.10	4.21	0.76	**1.00**	**[1.00,1.00]**	1.49	4.74	0.93
25-29	78	7.44	4.10	6.66	0.75	1.00	[0.51,1.95]	3.74	4.92	0.74
30-34	116	10.17	6.65	15.57	1.45	1.65	[0.75,3.63]	5.57	6.09	1.29
35-39	154	6.24	4.60	4.71	0.38	1.16	[0.75,1.78]	7.39	4.73	0.51
40-44	114	4.52	12.53	22.56	2.11	3.08	[1.54,6.18]	5.47	8.43	1.39
45-49	152	6.23	8.00	15.51	1.26	1.98	[1.14,3.45]	7.30	7.61	1.28
50-55	172	8.27	6.19	7.48	0.57	1.60	[1.03,2.49]	8.26	6.24	0.79
55-59	231	7.14	9.26	13.20	0.87	2.21	[1.26,3.86]	11.09	6.65	0.70
60-64	272	6.50	4.85	10.08	0.61	1.21	[0.75,1.95]	13.06	4.91	0.60
65-69	120	6.70	2.84	5.47	0.50	0.72	[0.43,1.18]	5.76	3.46	0.58
70-74	69	3.63	2.41	1.93	0.23	0.59	[0.38,0.93]	3.31	2.29	0.26
75+	77	5.25	3.00	3.19	0.36	0.72	[0.39,1.33]	3.70	2.55	0.32
Respondent sex (child or adult giving contact information)										
Male	909	49.79	4.79	7.87	0.26	**1.00**	**[1.00,1.00]**	43.64	5.18	0.29
Female	1167	49.92	6.58	12.73	0.37	1.35	[1.05,1.75]	56.02	5.56	0.31
Other	7	0.29	3.99	1.55	0.58			0.34	4.00	0.85
Type of day that interview was conducted on										
Weekday	1641	81.67	6.07	11.37	0.28	**1.00**	**[1.00,1.00]**	78.78	5.72	0.26
Weekend	442	18.33	3.93	5.83	0.28	0.65	[0.51,0.82]	21.22	4.14	0.34
Type of survey mode that interview was conducted in										
CAWI	1328	53.40	5.51	9.44	0.26	**1.00**	**[1.00,1.00]**	63.75	5.20	0.25
CATI	755	46.60	5.88	11.80	0.43	1.02	[0.77,1.35]	36.25	5.73	0.40

		Weighted	Number of contacts					Unweighted	Number of contacts	
	N	%	Mean	SD	SE	IRR	95% CI	%	Mean	SE
Household race										
Black or African American	64	6.31	8.39	17.70	2.21	1.42	[0.62,3.26]	3.07	6.52	1.95
Asian or Pacific Islander	79	5.10	6.41	9.20	1.04	1.08	[0.65,1.82]	3.79	4.85	0.73
White	1790	75.53	5.70	10.47	0.25	**1.00**	**[1.00,1.00]**	85.93	5.45	0.23
American Indian	53	1.01	3.86	4.90	0.67	0.73	[0.48,1.11]	2.54	4.06	0.65
Hispanic	70	7.25	3.68	3.72	0.44	0.67	[0.44,1.00]	3.36	4.39	0.59
Other or two or more races	27	4.81	4.37	9.15	1.76	0.78	[0.36,1.73]	1.30	5.22	1.92
Individual race										
Black or African American	41	3.68	4.40	5.35	0.84	0.71	[0.47,1.08]	2.00	4.17	1.14
Asian or Pacific Islander	55	3.62	5.86	8.59	1.16	0.96	[0.48,1.90]	2.68	4.49	0.76
White	1851	83.14	6.01	11.28	0.26	**1.00**	**[1.00,1.00]**	90.34	5.56	0.24
American Indian	37	0.80	3.37	2.89	0.48	0.60	[0.41,0.87]	1.81	3.41	0.50
Hispanic	40	5.22	2.87	2.91	0.46	0.48	[0.32,0.74]	1.92	3.65	0.62
Other or two or more races	10	1.46	9.793	15.69	4.96	1.82	[0.85,3.89]	0.49	9.00	4.96
Refused to Answer	49	3.84	2.44	2.36	0.34	0.39	[0.25,0.61]	2.35	3.20	0.35
Social contact survey household count										
1	326	11.50	4.11	9.18	0.51	**1.00**	**[1.00,1.00]**	15.65	4.28	0.58
2	629	29.60	4.88	9.55	0.38	1.18	[0.80,1.73]	30.20	5.02	0.42
3	347	14.61	5.09	6.72	0.36	1.21	[0.85,1.74]	16.66	5.11	0.37
4	501	23.36	6.29	11.91	0.53	1.55	[1.07,2.25]	24.05	6.11	0.47
5	195	14.06	8.31	15.47	1.11	1.99	[1.14,3.48]	9.36	6.69	0.74
6+	85	6.87	5.59	4.06	0.44	1.38	[0.92,2.06]	4.08	6.34	0.52
Super PUM										
Non-metro										
North Central MN	217	8.78	8.13	16.12	1.09	1.18	[0.76,1.85]	10.42	7.12	0.88
NE MN	266	7.56	7.19	14.91	0.91	0.99	[0.45,2.20]	12.77	5.56	0.71
Western MN	301	8.41	6.76	11.72	0.68	**1.00**	**[1.00,1.00]**	14.45	6.87	0.76
SE MN	136	9.23	5.89	6.88	0.59	0.82	[0.56,1.20]	6.53	5.51	0.53
Metro										
Outskirts of TC Suburbs	182	10.72	8.30	15.68	1.16	1.19	[0.73,1.94]	8.74	6.81	0.86
Anoka,Washington	171	10.93	5.70	7.79	0.60	0.84	[0.61,1.16]	8.21	5.47	0.57
Dakota,Scott,Carver	195	12.08	4.81	9.87	0.71	0.67	[0.39,1.14]	9.36	4.22	0.47
Ramsey	175	9.82	3.95	5.70	0.43	0.60	[0.43,0.84]	8.40	4.18	0.47
Hennepin	440	22.46	3.70	5.43	0.26	0.53	[0.40,0.70]	21.12	3.76	0.32

Values in bold represent the reference category in each regression

The sample excludes five observations with missing information on the number of interpersonal contacts. The sample assumes that children without reported contacts did have contacts with household members. In this table, we have not top-coded/censored the maximum number of contacts

### Survey questions

Respondents were asked to report their social contacts which occurred on the previous day. We adopted a similar definition of contacts as that used in the POLYMOD and many other contact studies [14, 22, 23]. A contact was defined as (1)** either** a two-way conversation with three or more words in the **physical presence** of another person **or** for children who are not yet speaking, a one-way conversation in the **physical presence** of another person; or (2)** physical skin-to-skin contact** (for example a handshake, hug, kiss or contact sports). These contacts are further classified as conversational or physical.

A key feature of the survey is that data were collected for both children and adults. Respondents were defined as adults who self-reported their contacts or children under age 18 whose contacts were reported by a household adult. All households with children under 18 were asked first to provide social contact information for a randomly selected child, followed by a request for the adult to continue and provide social contact data for themselves; consequently, some households had two respondents.

For each respondent in our survey, we collected information on their age. We then asked them to record the total number of contacts (overall and in school and work settings) on the previous day. Each survey participant was asked about contacts for a single day during the survey round. Then, for up to 30 of their interpersonal contacts, we collected information on the contact’s age, gender, the location of the contact (home, school, work, transportation, etc.), whether the contact was physical or not; the duration, and the frequency with which they were usually in contact with this person. Therefore, when we create the age-structured contact matrices, the number of contacts is top-coded/censored at 30. It is common to top-code/censor in these types of surveys. The POLYMOD surveys censored at 29 contacts [14, 22]. For respondents with a large number (10 or more) of school or work contacts, we collected aggregate information for the contacts in those settings. The survey and survey methodology are described in greater detail in Dorélien et al. [24].

### Data quality and sample selection

A random sample of 2790 households was drawn from MNHA for Round 1 of data collection. The response rate was 57 percent. Data was collected on 1602 households however 15 were dropped during data cleaning [24]. The final sample contained 1613 households with 2088 respondents. In our analysis, we excluded an additional five respondents that did not report (refused or don’t know) the total number of interpersonal contacts. The final sample consisted of 2083 participants (adults = 1594, children = 489).

For respondents with more than 10 interpersonal interactions at work (work outliers = 147 respondents), we did not collect detailed age information on their work contacts. Therefore, we impute the ages of those missing work contacts for work outliers. Because of top coding (an upward bound on the total number of contacts), we impute the ages of missing work contacts until a respondent has 30 detailed contacts. For instance, if a respondent provided detailed information about 10 contacts, but had 27 work contacts, we imputed the ages of the first 20 work contacts. Since we knew the total number of missing work contacts, we generated the age of each missing work contact by sampling contact’s ages from reported work contacts of other respondents who went to work, were in the same five-year age group and were of the same gender as the respondent. This assumes that people of the same age and gender interact with a similar mix of people in the workplace. The age distribution of people going to work during the SAH order was the same for the work outliers and those with fewer than 10 work contacts. Based on balance tests, the only variable that was predictive of being a work outlier was “female”. We assumed that people of the same age and gender interact with a similar mix of people in the workplace. As a sensitivity test, we also imputed missing work contacts using predictive mean matching (PMM) imputation. There were no statistically significant differences between the age groups of contacts generated by the two methods.

We also did not collect detailed school contact data for respondents (n = 17) with 10 or more contacts at school. We assumed that these contacts were of the same age as the respondents. During the data cleaning stage, we noticed that some (n = 76) of the child participants reported no contacts. We assumed that children without reported contacts did not have contacts outside the household but did still have contact with household members.

### Data analysis

In this paper, we pooled all contacts (physical and conversational) and did not restrict contacts based on duration, or frequency of reported contacts. We focused on describing and quantifying age patterns of social contacts to capture potential infectious disease transmission pathways between age groups and to provide critical input data for mathematical models of SARS-CoV-2 transmission [14].

### Descriptive analyses

Data cleaning and analysis were conducted using Stata version 16 [25] and the *socialmixr* R package [26]. First, we used histograms to show the distribution of mean daily contacts during the SAH. In Table 1, we tabulated the mean number of interpersonal contacts per day by respondent’s age group, gender, type of day, region, household race, individual race, and household size. [Household race (HHRACE) is a variable created for the weighting process and is an indicator of whether anyone in the MNHA household is identified with a specific race or ethnic identity. Since members of a household may have multiple races or ethnic groups, HHRACE uses a hierarchy system to categorize these households: Hispanic, other race or multi-racial, American Indian, Asian or Pacific Islander, Black, and White. For example, if one member reports being Hispanic, and all other report a different ethnicity, the household is categorized as Hispanic. But if no one reports being Hispanic or multi-racial in the household, and one reports being Asian (non-Hispanic), the household is categorized as Asian or Pacific Islander.] We display both the weighted and unweighted mean daily contacts, as well as the standard errors. To display variation, we also include the standard deviation. Finally, in Table 1, we also include the incidence rate ratio (IRR) estimated from a negative binomial regression that includes covariates for structural factors (survey mode and type of day). The number of contacts in the negative binomial regressions were not censored. We cluster the standard errors at the household level to account for multiple respondents from the same households. Our regression includes sampling weights [27]. We also tested whether time was correlated with the total number of social contacts, since behavior may change over time [28].

To understand how location influences the age pattern of contacts, we disaggregate the mean number of daily contacts by location and calculate the relative distribution of mean contacts in home, school, work, and other locations by respondent’s age group.

### Age-structured contact matrices (who interacts with whom)

We generated overall age-structured contact matrices as well as age-structured matrices by location using the *socialmixr* R package [30]. Data imputation was conducted before applying the *socialmixr* package because, by default, the package excludes contacts with missing or refused age information.

Respondents and their contacts were grouped into five or ten-year age groups and include individuals between the ages of 0 and 80; consequently, the raw contact matrix displays the mean number of daily contact (*M*_*ij*_) between respondents in age group *i* and their contacts in age group *j*. We also accounted for sampling weights, and weights for the type of day (a weight of 5 for the weekday and 2 for the weekend). We calculate *M*_*ij*_ as:

1 $${M}_{ij}= \frac{{\sum }_{t=1}^{{T}_{i}}{w}_{it}^{ds}{y}_{ijt}}{{\sum }_{t=1}^{{T}_{i}}{w}_{it}^{ds}}$$

where *w*_*it*_^*ds*^ is the weights for the type of day and sampling weights combined for respondent *t* in age group *i*, *y*_*ijt*_ is the reported number of contacts made by respondent *t* in age group *i* with a contact in age group *j* and *T*_*i*_ represents all respondents in age group *i*.

Because of differences in reporting, interpersonal contacts in our dataset are not reciprocal. Therefore, we use population data from the 2019 MN American Community Survey to make sure that at the population level the matrices are symmetrical/reciprocal [29]. This means that at a population level the total number of contacts made by respondents in age group *i* with contacts in age group *j* are the same as vice versa. We calculated the entries of the symmetric matrices (${M}_{ij}^{Symm}$) as:

2 $${M}_{ij}^{Symm}=\frac{{M}_{ij}{N}_{i}+{M}_{ij}{N}_{j}}{2{N}_{i}}$$

where *N*_*i*_ represents the sum of all individuals in age group *i* and age group *j* [2, 26, 29].

Finally, for each age-structured contact matrix, we also calculated a measure of age-assortativeness using the index *Q*. If individuals interact solely with others in their age group, then the *Q* index takes a value of one; if there is homogeneous mixing the *Q* index takes a value of zero [3, 30, 31]. The index is calculated by taking the trace of a matrix, *P*, whose elements represent the fraction of the **total contacts** of age group *i* with age group *j*, ${P}_{ij}={T}_{ij}/{\sum }_{j}{T}_{ij}$. The matrix ${T}_{ij}$ is obtained by multiplying ${M}_{ij}^{Symm}$ by a vector containing the number of people in each age group. The value of *n* represents the number of rows or columns of the *n* by *n* mixing matrix; in this study it is the number of age groups [3, 30, 32].

3 $$Q=\left[Tr\left(P\right)-1\right]/(n-1)$$

### Comparing matrices to published data and baselines (UK POLYMOD and ATUS home)

We compared our generated results to the UK POLYMOD matrices and calculated the percentage change in mean daily contacts. For our main analysis, we focused on a comparison with the UK POLYMOD matrices in predicting pre-SAH contact rates. We also compared the home location matrix with the ATUS pre-pandemic home matrix, which is representative of the contiguous US states and is based on data from 2003–2018 for respondents aged 15 and older [9] and calculated the difference and percentage change in mean daily contacts. We hypothesized that the mean number of household/home contacts would increase during the pandemic. We also compare our results to the synthetic US matrices derived from Prem et al. and calculate the percentage change in mean daily contacts (Additional file 1: Appendix SB) [11].

## Results

### Number of contacts

**Fig. 1:**
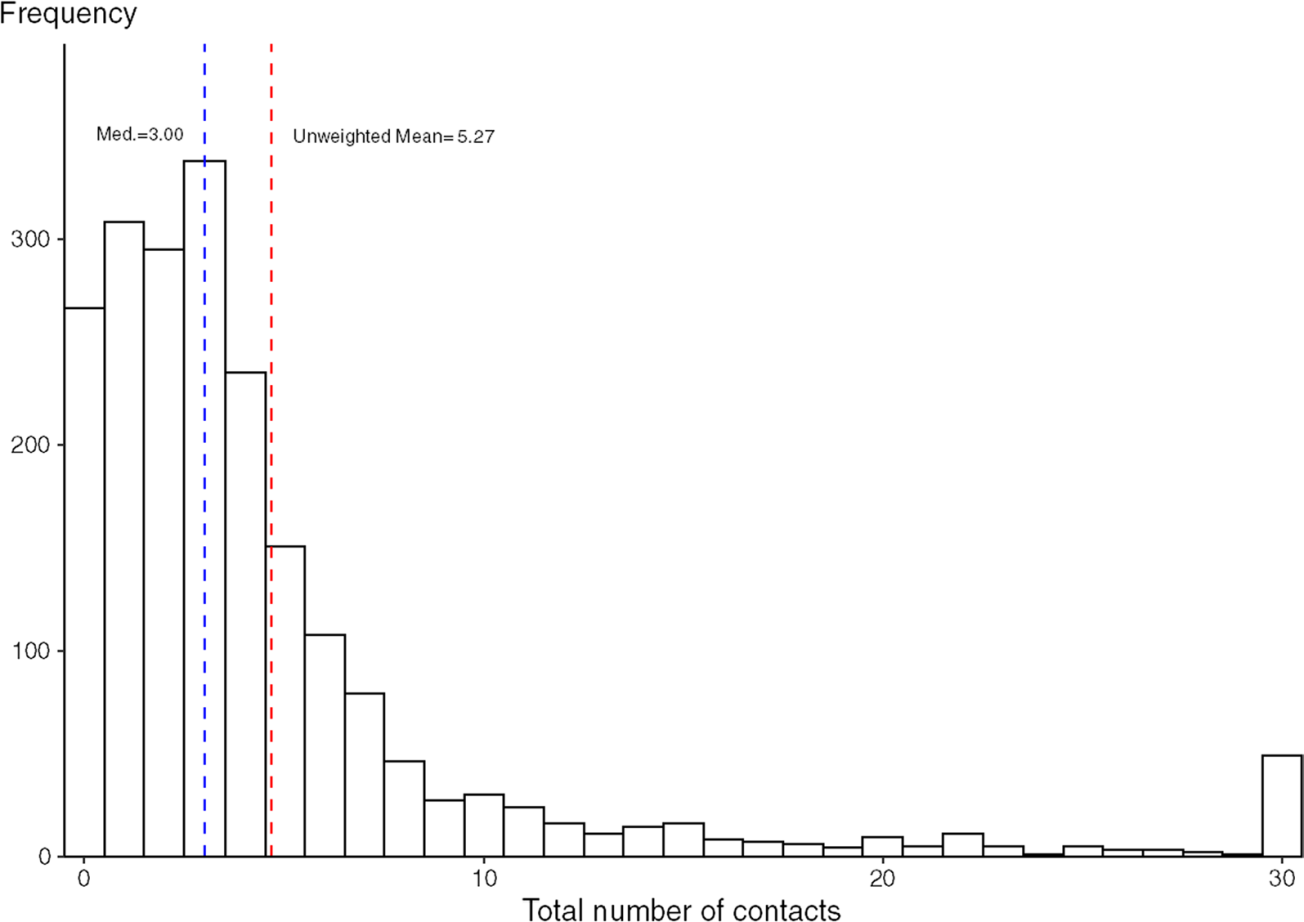
Histogram showing the distribution of the number of daily contacts. Maximum number of contacts has been top-coded at 30. Mean and median calculations are not based on top-coded data

The first survey responses (April 17, 2020) took place a month since the start of the stay home order (March 16, 2020) and 20 days after the more restrictive shelter in place order (SIPO) (March 28, 2020) [15]. The effects of the SAH and the SIPO did not result in any temporal differences in mean daily number of social contacts during the survey period. There was no correlation between the days since the start of our survey and the number of social contacts (each additional day was associated with 0.02 contacts which was not statistically significant). Consequently, Fig. 1 shows the histograms of reported contacts. The average number of daily contacts in the MN SCS Round 1 sample is 5.7 (5.4 if using unweighted data), however, the distribution of contacts is skewed (Skewness = 5.6 and Kurtosis = 40.3). The median number of daily contacts is 3.0; 82 percent of respondents reported six or fewer contacts (Fig. 1 and Table 1). There is a long tail with 45 (2.2%) respondents reporting more than 30 contacts and a handful reporting more than 100 contacts. Despite the stay-at-home order, 71 percent of the mean daily contacts were with non-household members.

### Demographic and structural factors associated with elevated contact rates

The 2083 participants in our analytic sample recorded a total of 10,983 unique contacts. Table 1 highlights the fact that the frequency of social contacts was not homogeneous across age, gender, race, region, or type of day. Additional file 1: Appendix SA contains a similar table but restricts contacts to those with non-household members. Based on the weighted means in Table 1, older teens (15–19 years old), and adults between the ages of 30–60 years had the highest number of daily contacts during the SAH order. Elderly participants (ages 65 +) had the fewest mean daily contacts yet more than 70 percent of their contacts were with non-household members; in contrast, for children below the age of 15, less than 50 percent of their contacts are with non-household members. On average, women had more daily contacts (6.6) compared to men (4.8) (IRR = 1.35; 95% CI = 1.05–1.75), and a higher share (74% versus 67%) of these contacts were with non-household members. Contacts were lower (3.9) during the weekend and less likely to be with non-household members compared to the mean of 6.1 daily weekday contacts (IRR = 0.66; 95% CI = 0.53–0.83). Except for the suburbs of the twin cities (TC) of Minneapolis and Saint Paul, the average number of daily contacts (and the proportion of contacts with non-household members) was lower in metro areas compared to non-metro areas. An additional analysis shown in Additional file 1: Appendix SB reveals that for respondents in the metro areas the majority of contacts took place at home while for those in non-metro areas the majority of contacts took place at work and school. The daily number of contacts tended to increase as household size increased from one to four; on the other hand, the share of contacts with non-household members declined with increasing household size. We had relatively few large (household size > 4) households therefore the confidence intervals were very large for these groups.

The way race/ethnicity was coded influenced patterns between groups. Between April 17th and May 17, 2020, in Minnesota, respondents in Black or African American households (which includes 26 White respondents living with Black household members) had approximately three (2.69) additional contacts (IRR = 1.42, 95% CI = 0.62–3.26) compared to respondents in White households. Seventy-eight percent of the Black or African American household contacts were with non-household members compared to 73 percent for White households. We find different results when we only consider the respondents self-reported race and do not the racial composition of the household. Non-Hispanic Black respondents had fewer contacts (mean = 4.40, IRR = 0.71, 95% CI = 0.47–1.08) compared to NH White respondents (mean = 6.01). This is driven by the fact that in our sample, NH White respondents living in households with black respondents had significantly more contacts (mean = 13.94) than NH-white respondents living in white households.

Respondents who self-reported as Asian or Pacific Islander or were classified as being in Asian/Pacific Islander households had approximately the same number of contacts as the respondent in White households (individual race IRR = 0.96, 95% CI = 0.48–1.90; household race IRR = 1.08; 95% CI = 0.65–1.82). Respondents in Hispanic households had significantly fewer contacts (mean = 3.68) compared to White households (IRR = 0.67; 95% CI = 0.44–1.00). The results were similar for respondents that self-reported their ethnicity as Hispanic (mean = 2.87; IRR = 0.48; 95% CI = 0.32–0.74). Likewise American Indians and respondents in American Indian households had fewer contacts compared to White respondents and White Households (individual race mean = 3.37, IRR = 0.60, 95% CI = 0.41–0.87; household race mean = 3.86, IRR = 0.73, 95% CI = 0.48–1.11). A smaller proportion of contacts were with non-household members for both Hispanic (40%) and American Indian (56%) respondents, this may be related to their household size. The number of respondents in the “other or two or more race” category was extremely small; therefore, the estimates may not be reliable.

### Location of contacts

**Fig. 2:**
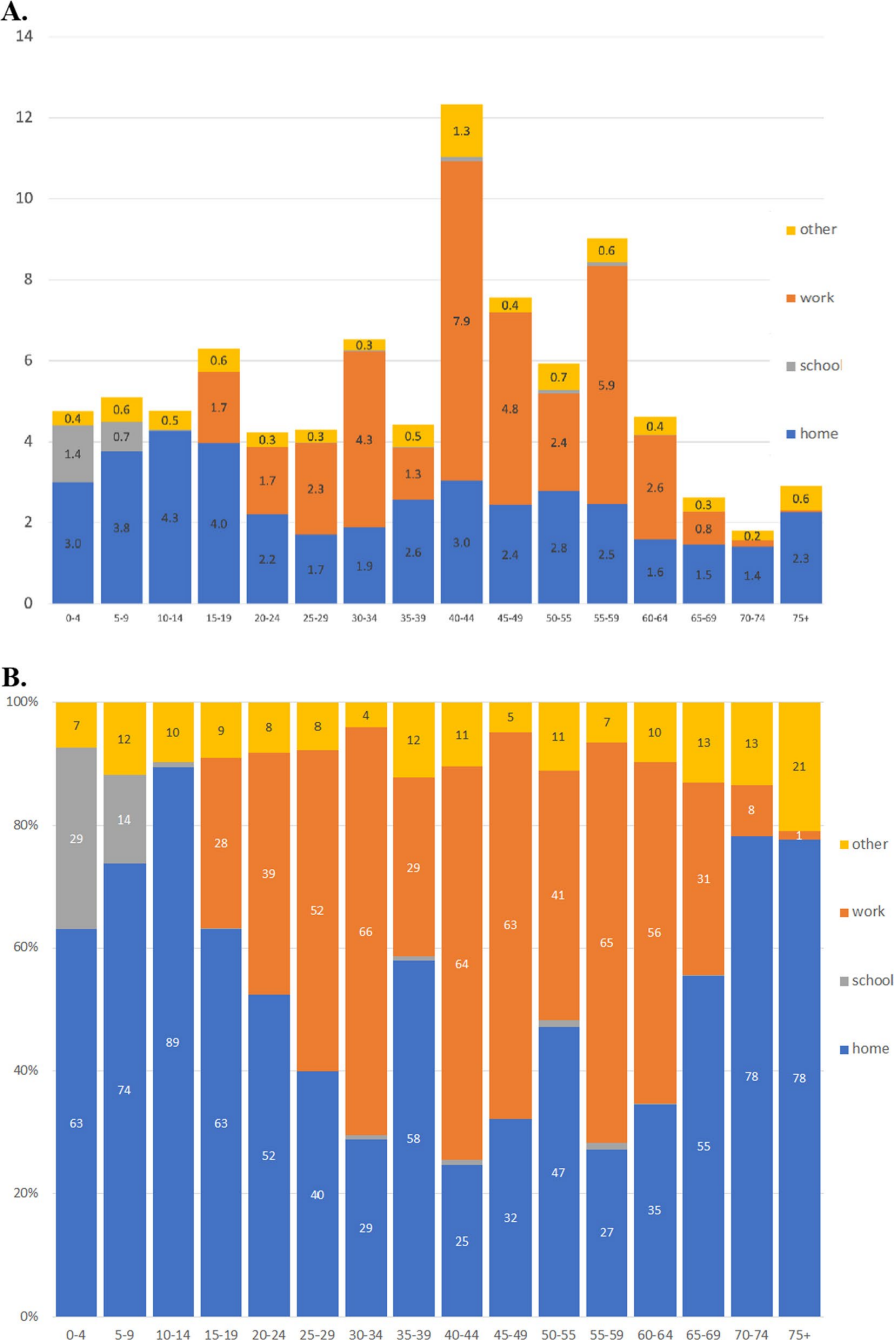
The age pattern of contacts by location, April 17-May 17, 2020. **A** Mean number of contacts by location and respondents age group. **B** Share of contacts (%) by location and respondents age group. For this analysis, the maximum number of contacts for a respondent was top coded at 30. The home category includes the respondent’s own home and someone else’s home. The mean number of contacts in panel A may not equal the values in Table 1 because these were based on the detailed contact data and not the total number of reported contacts. In the detailed contact data, a few contacts may appear in multiple locations. Sample weights were used

Figure 2 disaggregates the mean daily number of contacts by location (panel a) and displays the relative share (panel b) of contacts by location and respondent’s age group. For respondents younger than 25 and over 65, most contacts took place at the respondent’s home (or someone else’s home). Most contacts for respondents between the ages of 35–39 also took place in the home. This may be due to childcare duties during the SAH order. Children had a higher mean number of contacts taking place at home compared to adults, and adults aged 35–59 had a higher mean number of home contacts compared to adults 20–34 and 60 and older. The age pattern of home contacts was likely driven by differences in household composition over the life course. For working-age respondents (ages 15–69) approximately 30–66 percent of all contacts took place in the workplace. About 30% of all contacts for children under the age of five took place in a daycare setting during the SAH order.

### Age-structured contact matrices results

To generate the age-structured contact matrices the maximum number of contacts that an individual could have was restricted to 30. This is in line with the Mossong (2008) POLYMOD matrices where the maximum number of contacts was limited to 29 [14]. Therefore, for these analyses, the 2049 participants had 9618 unique contacts for which age was known or imputed.

**Fig. 3:**
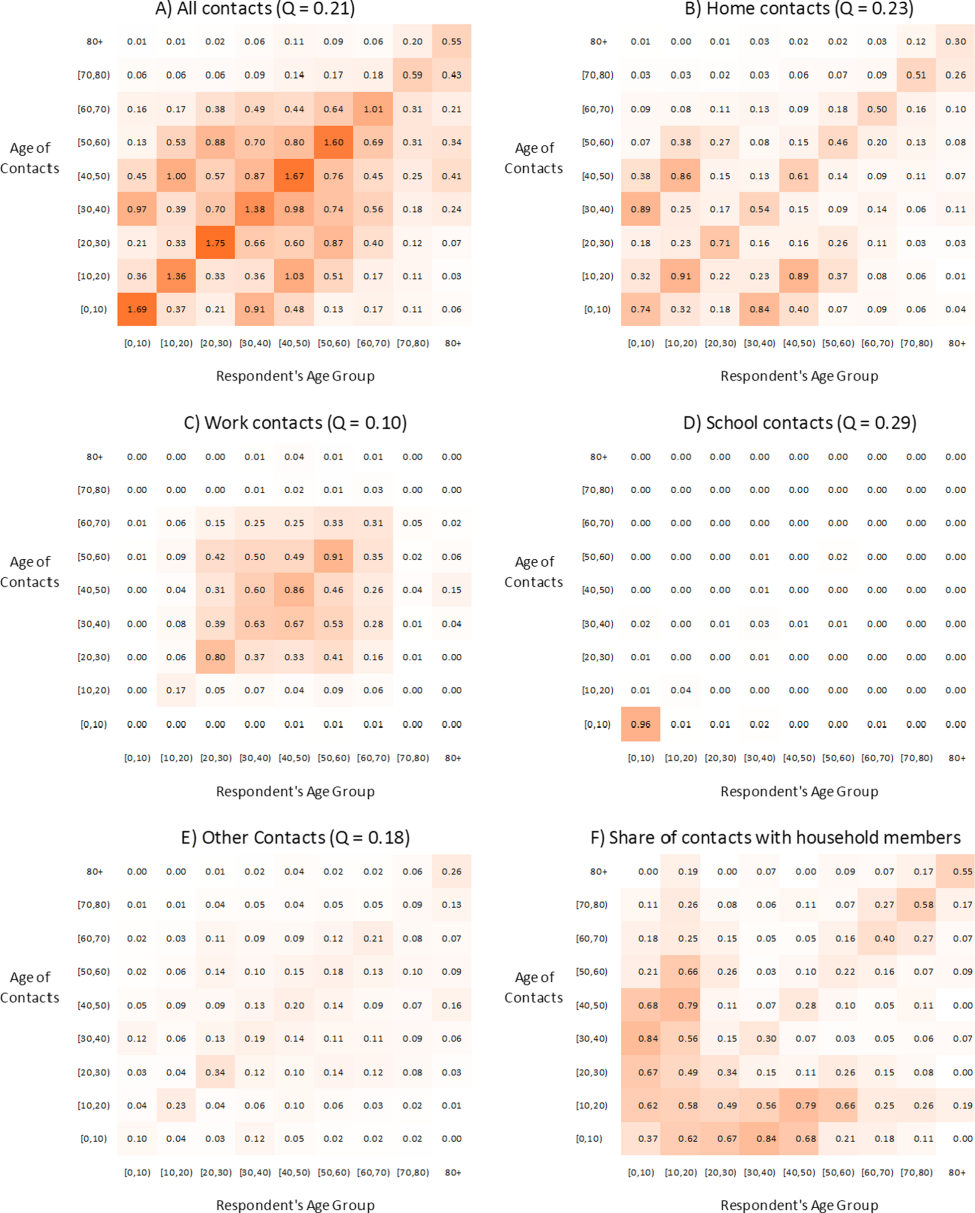
MN SCS Round 1 (April–May 2020) age-structured contact matrix during SAH Order by location. Home contacts were defined as own-home contacts, and other contacts included transport, store, outdoors, and other contacts not located at home, work, or school

Figure 3 presents the overall age-structured contact matrices as well as age-structured contact matrices for different locations. Overall, the age-assortative index *Q* of the SAH age-structured contact matrix measured in this study is equal to 0.21 (Fig. 3A); in comparison, the *Q* index for the pre-pandemic UK POLYMOD matrix is 0.18 (additional analysis would have to be conducted to determine whether the differences between the Q indices are statistically significant). The greatest amount of mixing took place between respondents and contacts in the same age group. However, unlike in pre-pandemic matrices such as the UK POLYMOD matrix, there was not much variation in the level of age-assortative contacts for those below age 60 (range is between 1.36 to 1.77 contacts). Children and young adults no longer had the largest number of assortative contacts (Additional file 1: Appendix SC1).

The SAH matrix also contains prominent parallel off-diagonal elements starting in the 30–40 years age groups for both respondents and contacts. As in the pre-pandemic matrices, these represent intergenerational mixing, especially between young children and adults at home (Fig. 3a, b). Most contacts between participants below age 50 and participants younger than 20 are between household members.

### Age mixing patterns by location

The location-specific age-structured contact matrices corroborate the fact that during the SAH order the greatest proportion of contacts took place at home (Fig. 3b) and for certain mixing patterns between age groups, contacts were dominated by contacts with household members (Fig. 3F). The matrices and corresponding *Q* indices also illustrate that the pattern of contacts across the age groups varied by setting. Contacts at home were dominated by interactions between parents and children as well as the interaction between people in the same age groups (Fig. 3b). The nature of contacts also differed based on the setting. Most physical contacts took place at home whereas contacts in other settings were primarily conversational (analysis not shown).

Work contacts were predominantly limited to individuals between 15 and 65 years old and were assortative by age (Fig. 3c). During the SAH order, schools were closed; however, some daycare facilities remained opened because childcare providers were considered an essential service [14]. Consequently, the “school” matrix shows that children below age five were mixing with other children (physical and conversational) and had contact with parents/guardians and childcare providers (Fig. 3d). There was a small number of contacts between adults, which most likely represent mixing between childcare providers and interactions between childcare providers and parents/guardians.

The “other” location matrix represents contacts that took place outside of home, school, or work. These contacts could take place while commuting, in stores, outdoors, and or in someone else’s home. The magnitude of contacts between people taking place in other locations is relatively low (Fig. 3e). Although age-assortative mixing is the most prominent mixing pattern in these settings, the difference when compared to interactions between people of different age groups was low (*Q* = 0.18). One of the most striking features of the “other” matrix was that people over 50 had almost no interactions with children under 10.

### Comparisons with pre-pandemic patterns: UK POLYMOD and US home matrix

If we assume that MN pre-pandemic contact patterns were like those of the United Kingdom, as measured in the UK POLYMOD survey then as a result of the pandemic and SAH order, the mean number of contacts shrunk for the entire population (with one exception) but there was also a change in age-based mixing patterns, therefore the reduction was not the same across every cell (Additional file 1: Appendix SC1). School children had a large reduction in the number of interpersonal contacts with other children and with young adults less than 30 years old (67–84% reduction). Children had a large absolute reduction especially with children their own ages (3–7 fewer contacts); this reflects the effects of school closure during the SAH. There was also a large reduction in contacts between non-institutionalized individuals aged 60 + years and those in younger age groups (on average a 70% reduction or 0.7 fewer contacts). The absolute reduction in the number of contacts for respondents aged 60 + years is not large because this age group has fewer contacts. The change in the mean number of contacts among adults between 20–60 years old was lower (on average 43% reduction). Many of these patterns are also evident if we compare the MN SAH matrix to the US pre-pandemic synthetic matrix from Prem, Cook, and Jit [11] (Additional file 1: Appendix SC2). The main difference is that when we compare with the pre-pandemic US synthetic matrix, we see an increase in the number of contacts between working-age respondents and those above 65; this is an artifact because the synthetic matrix assumed no work contacts for those above 65.

On average, there was an increase in the number of contacts taking place at home. However, the number of contacts at home varied during the SAH depending on the respondent’s age group. The number of contacts with others in the same age group increased (Additional file 1: Appendix SC3, see cells with black border). The number of home contacts between 65–75 years-old respondents and children 0–15 years old also increased; this could represent grandparents stepping in to provide childcare during the stay-at-home order, and combining of households to make this possible. The number of interactions between adults and children also increased, except for interactions with children 0–5 years old, which decreased. Overall, there appears to be a decline in the number of contacts between respondents younger than 55 and children between the ages of 0–5 years. Some of the declines in the number of contacts taking place in the home may be due to data quality issues. There is evidence that the MN SCS Round 1 survey may underestimate the number of household contacts. As mentioned in the materials and methods section, there were children for which parents stated that they had zero contacts; in the data cleaning process, we assumed that these children had contacts with household members. Additionally, there were still 85 adults who did not live alone yet reported zero contact with household members.

## Discussion

We conducted one of the first social contact surveys in the United States that includes children and includes a population that is representative at a state level. The age-structured contact matrices are a key input for modeling COVID-19 transmission dynamics and may also be useful for other respiratory infectious diseases such as influenza and pertussis.

During the stay-at-home orders implemented during the first wave of the COVID-19 pandemic, the mean number of contacts in MN was 5.6 (median = 3). Our results are in line with round 1 of the Berkeley Interpersonal Contact Survey (BICS), a non-probability sample of adults 18 + in 6 US cities conducted from April 10th to May 4th which also reported a median of three daily contacts [2]. If we assume that the pre-pandemic mean daily number of contacts was around 13, the POLYMOD countries average [14], then the lockdown significantly reduced the number of daily contacts. However, the average daily number of contacts in MN during the SAH order was higher than those in some other countries during their respective orders/lockdowns, which were more restrictive. For instance, the mean daily contacts during the United Kingdom’s lockdown was 2.8 daily contacts [1]; 3.2 in Luxembourg [33]; and 2.0 in Wuhan, China [34].

It is important to identify demographic groups that have high contact rates. Our results show that the mean number of social contacts during the SAH order was not homogeneous across region, race, gender, and age. We find some spatial differences in the mean number of daily contacts. During the first wave of the pandemic and SAH, on average respondents in non-metro areas had more contacts than those in the metro areas, and most of these contacts were at work or school. These spatial differences could be due to differences in occupation and respondent adherence to SAH orders. Another explanation is that during this period, COVID-19 cases were not as prevalent in non-metro settings [35].

The study found greater interpersonal contacts for Non-Hispanic Black **households** compared to Non-Hispanic White households, from which we can infer a greater likelihood of COVID-19 exposure. Exposure and rates of infections are of course also driven by the type of work, multigenerational households, and other factors. There is some evidence that workplace exposure maybe driving this result. In our sample, 40 percent of the employed respondents in NH Black households worked in the education and health care industry. We did not find the same pattern when we focused on respondent’s self-reported race, because the results were driven by White respondents living with Black household members. These White respondents were had very high number of contacts. Nevertheless, it is important to note that neither the values for NH Black households nor Black individuals were statistically different from NH White respondents. We have to be cautious about what we can conclude given that our sample of NH Black household and individuals were very small. In our survey, respondents from Hispanic households and those who self-identified as Hispanic had fewer interpersonal contacts; in our sample they were the group with the highest unemployment (including on layoff or furlough) rates. This study is limited in its ability to describe social contacts for people of color in MN; before adjusting for sampling weights, 86 percent of the respondents in the MN SCS sample were White households (Table 1). Consequently, the external validity of our findings for these groups may be limited. People of color in MN also have a different age distribution and are more likely located in metro areas. As such, future studies will need to oversample communities of color accounting for these differences to obtain more robust estimates of contacts in these communities. A deeper exploration of racial and ethnic differences in contact patterns is needed, as is an assessment of differences by employment status, occupation, income, and household composition.

The magnitude of contacts and the mixing patterns are not homogeneous by age. The regression analysis and age-structured matrices, both highlight the central role of individuals in the 40–45-year age group. Individuals in this age group had the highest mean number of contacts (weighted mean = 12.69); this is primarily driven by the presence of seven outliers with more than 75 work contacts. Nevertheless, this age group also had a relatively large number of contacts at home and in other locations (Fig. 2a). Individuals 40–50 years old have many age-assortative contacts in addition to contacts with people who are slightly older and younger (Fig. 3a). They also have a lot of contacts with individuals between the ages of 5–20 who are presumably their children (Table 1, Fig. 3). Importantly, this group appears to have had the smallest reduction in contacts during the SAH order (Fig. 2A, B); consequently 40–50 year olds may be an important group to study especially if they also are less likely to mask and have relatively high levels or susceptibility and infectiousness. Policymakers should also pay attention to individuals between 45–65 years (a.k.a. the squeezed/sandwich, generations who must take care of children and elderly parents) because those aged 70 + years and younger adults reported many contacts with this age group. While the large decrease in contacts between children and between those 60 + years and those in younger age groups likely helped reduce their COVID-19 infection risk there are downsides. The accompanying social isolation during COVID-19 pandemic has been found to be significantly associated with negative psychological effects including post-traumatic stress symptoms, confusion, and anger [36, 37].

While it is important to identify age groups that have high contact rates, it is also important to identify groups that may be the main drivers of disease transmission, since these may not always be the same. Disease transmission depends on the age pattern of contacts and the age pattern of susceptibility and infectiousness. One way to do this is to incorporate age-structured contact matrices in **models of infectious disease transmission**. The age-structured contact matrices generated in this paper have been used by the MN COVID-19 modeling team, a collaboration between the University of Minnesota School of Public Health and the Minnesota Department of Health. Specifically, it is possible to analyze how changes in contact rates impact the reproductive number (R0) of respiratory pathogens by comparing the dominant eigenvalues of the age-structured contact matrices [2]. Consequently, it is important to continuously measure contact patterns as they change, especially as different restrictions are lifted and as vaccines are rolled out to different age groups.

Our study has some important limitations. It is difficult to obtain detailed accurate information on all interpersonal contacts for respondents with large numbers of contacts. We capped the number of contacts respondents had to recall detailed information for at 30. We did this to limit the possibility of missing information and to reduce the burden on the respondent. To address this limitation, we collected information on the total number of contacts in different settings and developed a strategy to impute the missing reported ages at school and work for respondents with large numbers of contacts. The imputation likely added some uncertainty to some of the estimates of M_ij_. Another limitation might include self-selection into the sample; the respondents might be more compliant with executive orders. Nevertheless, the self-selection into the sample occurred before the start of the pandemic. There may be a lack of standardization in defining “effective contacts”; we did not take into consideration masking during the first round of data collection. We believe that our findings, despite this approach, are conservative for three reasons. First, there is some evidence that the survey may underestimate the number of household contacts; many respondents who lived with others reported having zero contacts. Social desirability bias could also be a factor due to respondents wanting to appear to comply with the SAH order. In this survey we asked people to recall their contacts, therefore respondents may forget to include some contacts. However, this error is likely small because respondents were asked to recall contacts from the previous day, and during the lockdown, most respondents had very few contacts [22, 38].

In conclusion, the MN SCS is an ongoing study monitoring social contact patterns in Minnesota during the COVID pandemic. It is one of the first surveys in the US to collect information on both children and adults. We have shown that during the SAH order there were large reductions in the number of daily contacts; and that there are significant differences in contact patterns based on respondents’ age, sex, gender, race/ethnicity, and region. We have also generated age-structured contact matrices which have been used in the MN COVID-19 modeling effort.

## Supplementary Information

**Additional file 1:** **Appendix SA. **Descriptive table for distribution of participants and daily non-household contacts by demographic characteristics**. Appendix SB. **Distribution of share of contacts by location across different Minnesota Regions. On average, regions with the fewest average daily contacts had a greater share of contacts taking place at home compared to regions with a high number of average daily contacts. **Appendix SC1. **Comparing UK POLYMOD and MN SCS Round 1 contact matrices. In the percentage change matrix, if there was an increase in the number of contacts during the SAH order, that value is coded in blue, if there was a decrease that value is in red. **Appendix SC2. **Comparing US Synthetic and MN SCS Round 1 contact matrices. In the percentage change matrix, if there was an increase in the number of contacts during the SAH order, that value is coded in blue, if there was a decrease that value is in red. **Appendix SC3.** ATUS vs MN Round 1 Home. In the percentage change matrix, if there was an increase in the number of contacts during the SAH order, that value is coded in blue, if there was a decrease that value is in red. Cells outlined in black represent interactions between respondents and contacts of the same age. **Appendix SD. **Negative binomial regression used to generate IRR and 95% confidence intervals in Table 1**.**

## Data Availability

The data is non-public and therefore cannot be shared at the individual level. We share the contact matrix data in the paper and upon request can share the corresponding confidence intervals that go with the estimated mean daily contacts between different age groups. The survey questionnaire is provided in the appendix of the Dorélien et al. [24] paper.
