# Supplementary material for: Quantifying social contact patterns in Minnesota during stay-at-home social distancing order
Source: BMC Infect Dis. 2023 May 15;23:324. doi: 10.1186/s12879-022-07968-1 (PMC10184106; doi:10.1186/s12879-022-07968-1)
Supplement: Supplementary file 1 — Additional file 1: Appendix SA. Descriptive table for distribution of participants and daily non-household contacts by demographic characteristics. Appendix SB. Distribution of share of contacts by location across different Minnesota Regions. On average, regions with the fewest average daily contacts had a greater share of contacts taking place at home compared to regions with a high number of average daily contacts. Appendix SC1. Comparing UK POLYMOD and MN SCS Round 1 contact matrices. In the percentage change matrix, if there was an increase in the number of contacts during the SAH order, that value is coded in blue, if there was a decrease that value is in red. Appendix SC2. Comparing US Synthetic and MN SCS Round 1 contact matrices. In the percentage change matrix, if there was an increase in the number of contacts during the SAH order, that value is coded in blue, if there was a decrease that value is in red. Appendix SC3. ATUS vs MN Round 1 Home. In the percentage change matrix, if there was an increase in the number of contacts during the SAH order, that value is coded in blue, if there was a decrease that value is in red. Cells outlined in black represent interactions between respondents and contacts of the same age. Appendix SD. Negative binomial regression used to generate IRR and 95% confidence intervals in Table 1. [file 12879_2022_7968_MOESM1_ESM.docx]

**Additional file 1**

**Appendix SA.** Descriptive table for distribution of participants and daily **non-household** contacts by demographic characteristics

**Notes:** The sample excludes five observations with missing information on the number of interpersonal contacts. The sample assumes that children without reported contacts did have contacts with household members. In this table, we have not top-coded the maximum number of contacts.

**Appendix SB.** Distribution of share of contacts by location across different Minnesota Regions. On average, regions with the fewest average daily contacts had a greater share of contacts taking place at home compared to regions with a high number of average daily contacts.

**Appendix SC1.** Comparing UK POLYMOD and MN SCS Round 1 contact matrices. In the percentage change matrix, if there was an increase in the number of contacts during the SAH order, that value is coded in blue, if there was a decrease that value is in red.

**Appendix SC2.** Comparing US Synthetic and MN SCS Round 1 contact matrices. In the percentage change matrix, if there was an increase in the number of contacts during the SAH order, that value is coded in blue, if there was a decrease that value is in red.

**Appendix SC3.** ATUS vs MN Round 1 Home. In the percentage change matrix, if there was an increase in the number of contacts during the SAH order, that value is coded in blue, if there was a decrease that value is in red. Cells outlined in black represent interactions between respondents and contacts of the same age.

**Appendix SD.** Negative binomial regression used to generate IRR and 95% confidence intervals in Table 1

**Negative binomial regression - Age Group**

| Total contacts | Coef. | | St.Err. | t-value | | p-value | [95% Conf | | Interval] | | Sig |
| --- | --- | --- | --- | --- | --- | --- | --- | --- | --- | --- | --- |
| 0-4 | 1.129 | | .25 | 0.55 | | .583 | .732 | | 1.741 | |  |
| 5-9 | 1.129 | | .241 | 0.57 | | .569 | .743 | | 1.715 | |  |
| 10-14 | 1.194 | | .235 | 0.90 | | .368 | .811 | | 1.757 | |  |
| 15-19 | 1.819 | | .553 | 1.97 | | .049 | 1.002 | | 3.3 | | ** |
| 20 -24 (reference) | 1 | | . | . | | . | . | | . | |  |
| 25-29 | 1.002 | | .341 | 0.01 | | .994 | .514 | | 1.954 | |  |
| 30-34 | 1.65 | | .664 | 1.25 | | .213 | .75 | | 3.629 | |  |
| 35-39 | 1.155 | | .253 | 0.66 | | .51 | .752 | | 1.775 | |  |
| 40-44 | 3.084 | | 1.095 | 3.17 | | .002 | 1.538 | | 6.184 | | *** |
| 45-49 | 1.983 | | .559 | 2.43 | | .015 | 1.141 | | 3.445 | | ** |
| 50-55 | 1.603 | | .359 | 2.11 | | .035 | 1.034 | | 2.486 | | ** |
| 55-59 | 2.207 | | .629 | 2.78 | | .005 | 1.263 | | 3.858 | | *** |
| 60-64 | 1.212 | | .294 | 0.79 | | .428 | .753 | | 1.949 | |  |
| 65-69 | .717 | | .183 | -1.30 | | .193 | .434 | | 1.184 | |  |
| 70-74 | .594 | | .136 | -2.27 | | .023 | .379 | | .931 | | ** |
| 75+ | .716 | | .226 | -1.06 | | .29 | .386 | | 1.329 | |  |
| cati | 1.003 | | .102 | 0.03 | | .973 | .822 | | 1.225 | |  |
| weekend | .661 | | .068 | -4.02 | | 0 | .541 | | .809 | | *** |
| Constant | 4.27 | | .866 | 7.16 | | 0 | 2.87 | | 6.354 | | *** |
|  | | | | | | | | | | | |
| Mean dependent var | | 5.389 | | | SD dependent var | | | 9.825 | |  |  |
| Pseudo r-squared | | 0.028 | | | Number of obs | | | 2083 | |  |  |
| Chi-square | | 93.538 | | | Prob > chi2 | | | 0.000 | |  |  |
| Akaike crit. (AIC) | | 29989963.097 | | | Bayesian crit. (BIC) | | | 29990070.287 | |  |  |
| *Note: *** p<.01, ** p<.05, * p<.1 The reference group for weekend is weekday, and the reference group for survey mode cati is cawi. Type of day and type of survey mode in this regression serve as covariates.* | | | | | | | | | | | |

**Negative binomial regression - Gender**

| Total contacts | Coef. | | St.Err. | t-value | | p-value | [95% Conf | | Interval] | | Sig |
| --- | --- | --- | --- | --- | --- | --- | --- | --- | --- | --- | --- |
| Male (reference) | 1 | | . | . | | . | . | | . | |  |
| Female | 1.354 | | .179 | 2.29 | | .022 | 1.045 | | 1.754 | | ** |
| Something else | .783 | | .105 | -1.82 | | .069 | .601 | | 1.02 | | * |
| cati | 1.026 | | .139 | 0.19 | | .85 | .787 | | 1.338 | |  |
| weekend | .664 | | .074 | -3.65 | | 0 | .533 | | .827 | | *** |
| Constant | 5.08 | | .482 | 17.12 | | 0 | 4.217 | | 6.119 | | *** |
|  | | | | | | | | | | | |
| Mean dependent var | | 5.389 | | | SD dependent var | | | 9.825 | |  |  |
| Pseudo r-squared | | 0.007 | | | Number of obs | | | 2083 | |  |  |
| Chi-square | | 18.807 | | | Prob > chi2 | | | 0.001 | |  |  |
| Akaike crit. (AIC) | | 30626407.693 | | | Bayesian crit. (BIC) | | | 30626441.543 | |  |  |
| *Note: *** p<.01, ** p<.05, * p<.1 The reference group for weekend is weekday, and the reference group for survey mode cati is cawi. Type of day and type of survey mode in this regression serve as covariates.* | | | | | | | | | | | |
|  | | | | | | | | | | | |
|  | | | | | | | | | | | |

**Negative binomial regression - Weekend and Survey Mode**

| Total contacts | Coef. | | St.Err. | t-value | | p-value | [95% Conf | | Interval] | | Sig |
| --- | --- | --- | --- | --- | --- | --- | --- | --- | --- | --- | --- |
| Weekend | .649 | | .077 | -3.65 | | 0 | .514 | | .819 | | *** |
| cati | 1.018 | | .147 | 0.12 | | .904 | .766 | | 1.351 | |  |
| Constant | 6.021 | | .61 | 17.72 | | 0 | 4.937 | | 7.343 | | *** |
|  | | | | | | | | | | | |
| Mean dependent var | | 5.389 | | | SD dependent var | | | 9.825 | |  |  |
| Pseudo r-squared | | 0.004 | | | Number of obs | | | 2083 | |  |  |
| Chi-square | | 13.459 | | | Prob > chi2 | | | 0.001 | |  |  |
| Akaike crit. (AIC) | | 30730418.816 | | | Bayesian crit. (BIC) | | | 30730441.382 | |  |  |
| *Note: *** p<.01, ** p<.05, * p<.1 The reference group for weekend is weekday, and the reference group for survey mode cati is cawi. We examine the correlation between total contacts and type of day and type of survey mode in this regression.* | | | | | | | | | | | |

**Negative binomial regression - Household Race**

| Total contacts | Coef. | | St.Err. | t-value | | p-value | [95% Conf | | Interval] | | Sig |
| --- | --- | --- | --- | --- | --- | --- | --- | --- | --- | --- | --- |
| White (reference) | 1 | | . | . | | . | . | | . | |  |
| Black | 1.421 | | .603 | 0.83 | | .407 | .619 | | 3.264 | |  |
| Asian or Pacific | 1.084 | | .287 | 0.30 | | .761 | .645 | | 1.823 | |  |
| Other or two or more | .784 | | .316 | -0.60 | | .546 | .356 | | 1.726 | |  |
| American Indian | .731 | | .156 | -1.47 | | .141 | .481 | | 1.11 | |  |
| Hispanic | .665 | | .137 | -1.98 | | .048 | .444 | | .996 | | ** |
| cati | 1 | | .127 | -0.00 | | .998 | .78 | | 1.281 | |  |
| weekend | .676 | | .082 | -3.22 | | .001 | .533 | | .858 | | *** |
| Constant | 6.067 | | .624 | 17.54 | | 0 | 4.96 | | 7.421 | | *** |
|  | | | | | | | | | | | |
| Mean dependent var | | 5.389 | | | SD dependent var | | | 9.825 | |  |  |
| Pseudo r-squared | | 0.007 | | | Number of obs | | | 2083 | |  |  |
| Chi-square | | 18.518 | | | Prob > chi2 | | | 0.010 | |  |  |
| Akaike crit. (AIC) | | 30628154.652 | | | Bayesian crit. (BIC) | | | 30628205.426 | |  |  |
| *Note: *** p<.01, ** p<.05, * p<.1 The reference group for weekend is weekday, and the reference group for survey mode cati is cawi. Type of day and type of survey mode in this regression serve as covariates.* | | | | | | | | | | | |
|  | | | | | | | | | | | |

**Negative binomial regression - Individual Race**

| Total contacts | Coef. | | St.Err. | t-value | | p-value | [95% Conf | | Interval] | | Sig |
| --- | --- | --- | --- | --- | --- | --- | --- | --- | --- | --- | --- |
| American Indian | .597 | | .114 | -2.70 | | .007 | .41 | | .868 | | *** |
| Asian | .955 | | .336 | -0.13 | | .895 | .479 | | 1.902 | |  |
| Black | .708 | | .151 | -1.61 | | .107 | .466 | | 1.077 | |  |
| Hispanic | .484 | | .104 | -3.37 | | .001 | .317 | | .737 | | *** |
| White (reference) | 1 | | . | . | | . | . | | . | |  |
| Other or two or more | 1.82 | | .706 | 1.54 | | .123 | .851 | | 3.893 | |  |
| refuse | .389 | | .088 | -4.19 | | 0 | .25 | | .605 | | *** |
| cati | 1.036 | | .139 | 0.26 | | .795 | .795 | | 1.348 | |  |
| weekend | .631 | | .073 | -3.97 | | 0 | .502 | | .792 | | *** |
| Constant | 6.347 | | .628 | 18.69 | | 0 | 5.229 | | 7.704 | | *** |
|  | | | | | | | | | | | |
| Mean dependent var | | 5.389 | | | SD dependent var | | | 9.825 | |  |  |
| Pseudo r-squared | | 0.012 | | | Number of obs | | | 2083 | |  |  |
| Chi-square | | 41.036 | | | Prob > chi2 | | | 0.000 | |  |  |
| Akaike crit. (AIC) | | 30482272.831 | | | Bayesian crit. (BIC) | | | 30482329.246 | |  |  |
| *Note: *** p<.01, ** p<.05, * p<.1 The reference group for weekend is weekday, and the reference group for survey mode cati is cawi. Type of day and type of survey mode in this regression serve as covariates.* | | | | | | | | | | | |

**Negative binomial regression - Household Size**

| Total contacts | Coef. | | St.Err. | t-value | | p-value | [95% Conf | | Interval] | | Sig |
| --- | --- | --- | --- | --- | --- | --- | --- | --- | --- | --- | --- |
| 1 (reference) | 1 | | . | . | | . | . | | . | |  |
| 2 | 1.178 | | .231 | 0.83 | | .404 | .802 | | 1.729 | |  |
| 3 | 1.212 | | .222 | 1.05 | | .295 | .846 | | 1.735 | |  |
| 4 | 1.55 | | .294 | 2.30 | | .021 | 1.068 | | 2.249 | | ** |
| 5 | 1.987 | | .568 | 2.40 | | .016 | 1.135 | | 3.479 | | ** |
| 6 | 1.375 | | .283 | 1.55 | | .121 | .919 | | 2.057 | |  |
| cati | .981 | | .124 | -0.15 | | .88 | .765 | | 1.258 | |  |
| weekend | .646 | | .078 | -3.63 | | 0 | .51 | | .818 | | *** |
| Constant | 4.454 | | .703 | 9.47 | | 0 | 3.27 | | 6.069 | | *** |
|  | | | | | | | | | | | |
| Mean dependent var | | 5.389 | | | SD dependent var | | | 9.825 | |  |  |
| Pseudo r-squared | | 0.010 | | | Number of obs | | | 2083 | |  |  |
| Chi-square | | 18.198 | | | Prob > chi2 | | | 0.011 | |  |  |
| Akaike crit. (AIC) | | 30528323.960 | | | Bayesian crit. (BIC) | | | 30528374.734 | |  |  |
| *Note: *** p<.01, ** p<.05, * p<.1 The reference group for weekend is weekday, and the reference group for survey mode cati is cawi. Type of day and type of survey mode in this regression serve as covariates.* | | | | | | | | | | | |

**Negative binomial regression - Region**

| Total contacts | Coef. | | St.Err. | t-value | | p-value | [95% Conf | | Interval] | | Sig |
| --- | --- | --- | --- | --- | --- | --- | --- | --- | --- | --- | --- |
| Western MN (reference) | 1 | | . | . | | . | . | | . | |  |
| NE MN | .994 | | .403 | -0.01 | | .988 | .449 | | 2.2 | |  |
| North Central MN | 1.18 | | .269 | 0.73 | | .468 | .755 | | 1.846 | |  |
| Outskirts of TC Suburbs | 1.185 | | .297 | 0.68 | | .499 | .725 | | 1.936 | |  |
| Anoka, Washington | .842 | | .136 | -1.07 | | .287 | .614 | | 1.155 | |  |
| Dakota, Scott, Carver | .67 | | .183 | -1.47 | | .141 | .392 | | 1.143 | |  |
| Hennepin | .53 | | .074 | -4.56 | | 0 | .404 | | .696 | | *** |
| Ramsey | .599 | | .104 | -2.95 | | .003 | .426 | | .842 | | *** |
| SE MN | .818 | | .159 | -1.04 | | .3 | .559 | | 1.197 | |  |
| cati | 1.022 | | .119 | 0.19 | | .852 | .813 | | 1.285 | |  |
| weekend | .655 | | .068 | -4.05 | | 0 | .534 | | .804 | | *** |
| Constant | 7.348 | | .883 | 16.60 | | 0 | 5.806 | | 9.3 | | *** |
|  | | | | | | | | | | | |
| Mean dependent var | | 5.389 | | | SD dependent var | | | 9.825 | |  |  |
| Pseudo r-squared | | 0.017 | | | Number of obs | | | 2083 | |  |  |
| Chi-square | | 51.253 | | | Prob > chi2 | | | 0.000 | |  |  |
| Akaike crit. (AIC) | | 30332140.802 | | | Bayesian crit. (BIC) | | | 30332208.501 | |  |  |
